# Effect of Infill Parameters on the Compressive Strength of 3D-Printed Nylon-Based Material

**DOI:** 10.3390/polym15020255

**Published:** 2023-01-04

**Authors:** Jingjing Liu, Muhammad Awais Naeem, Mouaz Al Kouzbary, Hamza Al Kouzbary, Hanie Nadia Shasmin, Nooranida Arifin, Nasrul Anuar Abd Razak, Noor Azuan Abu Osman

**Affiliations:** 1 Centre for Applied Biomechanics, Department of Biomedical Engineering, Faculty of Engineering, University of Malaya, Kuala Lumpur 50603, Malaysia; 2 The Chancellery, Universiti Tenaga Nasional, Kajang 43000, Malaysia

**Keywords:** 3D print, FDM, carbon fiber-reinforced nylon, compressive strength, infill pattern, infill density, powered ankle-foot prosthesis

## Abstract

3D printing is the most suitable method to manufacture the frame parts of powered ankle-foot prostheses but the compressive strength of the 3D-printed part needs to be ensured. According to the compression test standard ASTM D695, the effect of infill pattern and density, which is transferred to the mass of the standard specimen, on the compressive strength is investigated with a carbon fiber-reinforced nylon material. With the same infill pattern, specimens with more mass have a higher compressive strength. With the same mass, specimens with triangular fill have a higher compressive strength than those with rectangular and gyroid fills. Compared with specimens with a solid fill, specimens with a triangular fill can also provide more compressive strength in a unit mass. According to the results of standard specimens, following the requirement of strength and lightweight, 41% triangular fill is selected to manufacture the supporting part of a powered ankle-foot prosthesis. Under a compressive load of 1225 N, the strain of the assembly of the standard adaptor and the 3D-printed part is 1.32 ± 0.04%, which can meet the requirement of the design. This study can provide evidence for other 3D-printed applications with the requirement of compressive strength.

## 1. Introduction

Three-dimensional (3D) printing, one of the additive manufacturing methods, has been well-known and widely employed in industry. Compared with conventional manufacturing methods, the advantages of 3D printing include, but are not limited to, the high flexibility of design, ease of fabricating complex structures, and reduction in material waste [1]. The most common method of 3D printing is fused deposition modeling (FDM), also known as fused filament fabrication (FFF). In the FDM/FFF technology, the raw materials are usually continuous filaments of thermoplastic polymer or composites. One or more filaments are heated by the extruder to a semi-liquid state, printed on the base plate layer by layer, and solidified again at room temperature to form the designed structure [2].

Several kinds of materials can be manufactured by the FDM/FFF method. Thermoplastic polymers are the most common, such as acrylonitrile butadiene styrene (ABS), polylactic acid (PLA, a degradable bioplastic), polyamide (also known as nylon), and polyether ether ketone (PEEK) [3]. The thermoplastic polymers are also fiber-reinforced into composites with carbon or glass fiber to enhance their mechanical properties. In addition, some studies also report the utilization of low-melting-temperature metal alloys or metal-polymer composites in FDM/FFF printing [4]. Due to the above advantages and materials, nowadays, 3D printing performs outstandingly in rapid conceptual or customized prototyping.

A powered ankle-foot prosthesis is a kind of active medical device that can help below-knee amputees recover their functional mobility. Because the powered ankle-foot prosthesis can provide net power during the amputees’ daily activities, which is the capacity that the traditional prostheses do not have, wearing the powered ankle-foot prosthesis makes it possible to completely recover amputees’ normal gait. According to the statistics, more than 90 different powered ankle-foot prostheses have been developed since 2000 [5]. Because of the personalized demands of amputees in daily activities, it is believed that the selectable modular design of powered ankle-foot prostheses is the direction of future products. For the modular design of a powered ankle-foot prosthesis, the frame parts usually possess the abovementioned features of complex structure and customization. Therefore, 3D printing should be the most appropriate method to manufacture these mechanical parts. However, the most critical issue from rapid prototyping to final functional parts is ensuring the mechanical properties of 3D-printed products.

For regular 3D printers, various printing parameters, such as layer thickness, infill pattern, infill density, and build orientation, can be set. For some printers with a high degree of autonomy, additional parameters include print speed, nozzle temperature, chamber temperature, plate temperature, cooling fan speed, and so on. Previous researchers have investigated the effect of some printing parameters on the mechanical properties of 3D-printed parts [6,7,8]. For the application of powered ankle-foot prostheses, the 3D-printed frame parts are mainly used to support the user’s weight. Therefore, the manifestation of the load includes compressing, bending, and shearing loads. The required bending and shearing strengths can be achieved by increasing the corresponding size of the structures. However, for the required compressive strength, the only way is to investigate the compressive strength provided by 3D-printed parts with different printing parameters because the contact area with some standard parts cannot be increased. Compared with the degree of the influence of microscale, which is usually affected by the parameters such as the print speed or temperature, on the mechanical properties of 3D printed parts, differences in the macroscopic structure are more likely to significantly influence the compressive strength.

There is no doubt that infill patterns and densities are the parameters that significantly directly influence the macroscopic internal structure of 3D-printed parts. For the mechanical properties of 3D-printed parts, the effect of infill density has been widely investigated with different combinations of other 3D printing parameters. The summary of the investigated 3D printing parameters, materials, and mechanical properties in the literature is listed in Table 1.

Only focusing on the effect of infill density on mechanical properties, results from the literature are consistent. That is, more infill density can improve the mechanical properties of 3D-printed parts when other 3D printing parameters are reasonable and the same. In addition, according to the literature, some 3D printing parameters have an interaction or moderating effect on the above influence of the infill density, but others do not.

Including infill density, the build orientation, which refers to the inclination of the part on the build platform with respect to the X, Y, and Z axes (side, flat, and up) [18], is also studied in [10,11,16]. The results show that a non-horizontal build orientation reduces the tensile strength of the selected materials [10,16], and especially when the build orientation is along the Z-axis, the increase in infill pattern cannot improve the tensile strength anymore [11]. Layer thickness is one crucial factor, and it moderates the infill density’s influence on mechanical properties. However, for different mechanical properties, the effect is also different. When the layer thickness increases, the increase in infill density provides less improvement in the flexure strength of PLA or PLA-based materials [9,14]. Conversely, when the layer thickness increases, the influence of infill density on the tensile strength is enhanced [14,15]. Raster angle, which refers to the direction of the raster with respect to the main axis of the 3D-printed part [19], does not express a clear relationship with the influence of the infill pattern on mechanical properties [9,12]. Similarly, the relationship between extruder temperature and the influence of the infill density on mechanical properties is ambiguous [15,16].

Some literature also considers the infill pattern and infill density together, and the 3D printing parameters, materials, and mechanical properties studied in the literature are listed in Table 2.

In [20], the selected five parameters are tested independently. The results of infill density are consistent with the above conclusion. Under 40% infill density, the tensile strength provided by linear and rectilinear infill patterns is about 85% of those by honeycomb, concentric, and top concentric patterns. Similarly, with the independent test of three parameters, the infill pattern of wiggle is the best with 100% infill density [21]. Considering the interaction between the infill pattern and density, the results of [22,26,27] show that the performance of the infill pattern is different under different infill densities. For example, when the infill density is 100%, the tensile and flexural strengths with the concentric pattern are considerably higher than those with a ±45-degree linear pattern. However, the difference is not significant with 60% infill density [22]. The reference [23] contributes a lot to a similar topic, where 121 sets with different infill patterns (13 kinds) and densities (from 10% to 90%) are compared. Among all the sets, the concentric pattern with 90% can provide the maximal tensile strength, and the speed of the increase in tensile strength with the concentric pattern is also fastest with the increase in infill density. Due to the different range of infill patterns, the results from other studies are not quite the same. A triangular pattern is reported to be better than grid and cubic patterns [24]. Among the infill patterns of honeycomb, wiggle, grid, and rectilinear, honeycomb and grid patterns are almost the same and also the best [25].

However, most of the above research focused on the tensile strength of the materials, including a few on impact and flexural strengths. Only a few studies related to the effect of 3D printing parameters on compressive strength. The references [29,30] study the influence of two kinds of build orientations (horizontal and vertical) and four kinds of 3D printing styles (low, high, double dense, and solid) from Insight^®^ software on the compressive strength of 3D-printed ABS specimens, respectively. The former result shows that, under the given 3D printing parameters, the compressive strength of the horizontally printed specimens is higher than that of the vertically printed specimens [29]. Based on the material of PLA, it is found that the infill density has a positive influence on the maximal compressive strength [31,32,33], and the layer thickness and print speed do not have a significant influence on this effect [31]. The effect of infill patterns and densities on the compressive yield strength of PLA specimens is investigated [34]. The results show that all infill patterns perform almost the same with 100% density; the triangular pattern is the best with 20% and 60% densities; and diamond and hexagonal patterns are the best with 40% and 80% densities, respectively.

Towards the abovementioned application, that is, 3D-printed frames of powered ankle-foot prostheses, this study will further study the effect of infill patterns and densities on the compressive strength based on a kind of carbon fiber-reinforced nylon material. Next, considering that the 3D-printed frame parts of powered ankle-foot prostheses should be as light as possible, the selection of an appropriate parameter set of infill pattern and density for a functional part is completed and verified.

## 2. Materials and Methods

### 2.1. Preparation and Test of Standard Specimens

Standard specimens for compressive strength tests are printed by the Mark Two desktop 3D printer (Markforged, Watertown, MA, USA) with the material named Onyx^TM^ (Markforged, Watertown, MA, USA), which is a kind of fiber-reinforced composite base material that is a micro-carbon-fiber-filled nylon. The size of the specimens follows the ASTM standard D695, which is 12.7 mm in diameter by 25.4 mm (cylinder).

Because of the limitation of the official cloud-based slicing software, Eiger^TM^ (Markforged, Watertown, MA, USA), only three kinds of infill patterns, triangular, rectangular, and gyroid fills, can be compared to a solid infill pattern. The solid fill should have the maximal compressive strength, which will be the reference. In Eiger^TM^, the ranges of infill density for each infill pattern are also limited, specifically 28% to 55% for a triangular fill, 0% to 92% for a rectangular fill, and 28% to 52% for a gyroid fill, with the resolution of adjusting the infill density being 1% in the corresponding range. Considering that one of the aims of this study is to select proper 3D printing infill parameters to reduce the weight of the 3D-printed frames of powered ankle-foot prostheses, the infill density is transferred to the mass of specimens as a basis for grouping and comparison. Compressive specimens with different combinations of fill densities and infill patterns are printed and weighed by a digital scale whose accuracy is 0.01 g. Finally, we selected the specimens with different infill patterns but the same mass after rounding, which are listed in Table 3.

In terms of other 3D-printing parameters, the layer thickness is 0.1 mm; the number of wall layers is set to 1 layer (0.4 mm, which is identical to the output diameter of the nozzle), and that of roof & floor layers is also set to 1 layer (0.1 mm) to minimize the influence from the wall and the roof and floor on the compressive strength; and the extruder temperature is 275 °C. Standard specimens with each set of the above parameters are printed five times independently according to the requirement of D695.

**Figure 1:**
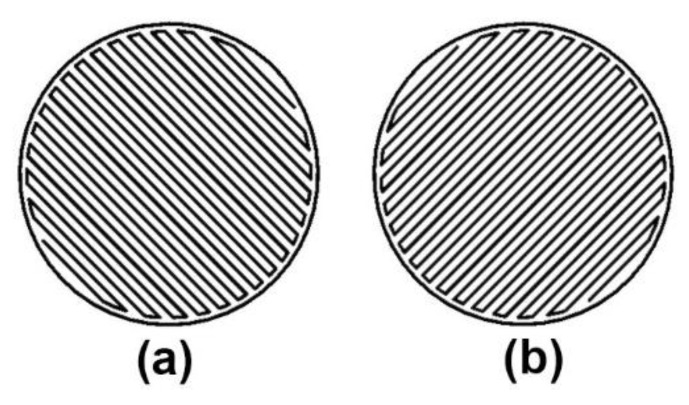
Tracing patterns of roof and floor layers. (**a**) roof layer; (**b**) floor layer.

For each infill pattern, the tracing patterns of the roof and floor layers are the same, as shown in Figure 1.

**Figure 2:**
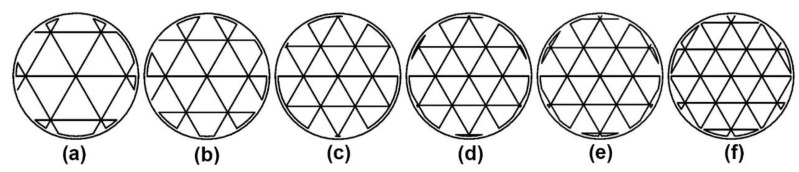
Tracing patterns of triangular fill with different infill densities. (**a**) 28%; (**b**) 34%; (**c**) 41%; (**d**) 42%; (**e**) 43%; (**f**) 46%.

For the middle layers, it is worth noting that the 3D printing program for each infill pattern is inconsistent. Firstly, the triangular fill is the simplest program, which repeats the unitary tracing pattern, shown in Figure 2.

**Figure 3:**
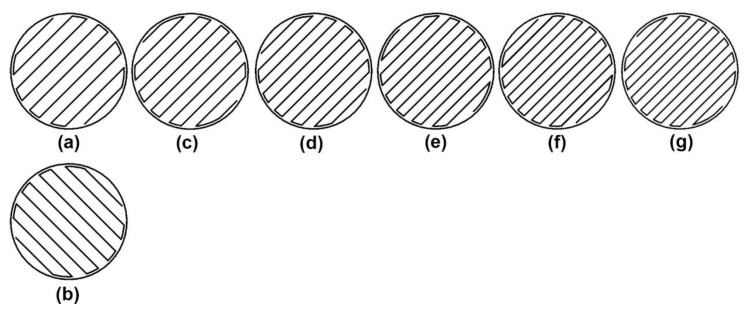
Tracing patterns of rectangular fill with different infill densities. (**a**) even layers for 31%; (**b**) odd layers for 31%; (**c**) odd layers for 35%; (**d**) odd layers for 38%; (**e**) odd layers for 41%; (**f**) odd layers for 45%; (**g**) odd layers for 48%.

Secondly, the rectangular fill follows two tracing patterns which rotate 90 degrees to each other. Take 31% rectangular fill, where the even layers follow Figure 3a and the odd layers (starting from the third layer) follow Figure 3b. The odd-layer tracing patterns of other infill densities are also shown in Figure 3.

Thirdly, the solid fill can be considered a particular rectangular fill whose infill density is 100%. Therefore, the odd layers follow the same tracing patterns as the floor layer (Figure 1b), and the even layers follow that of the roof layer (Figure 1a).

**Figure 4:**
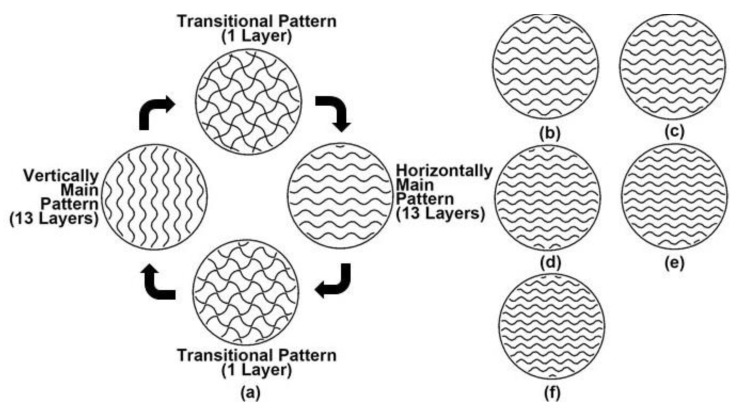
Tracing patterns of gyroid fill with different infill densities. (**a**) complete cycle for 36%; (**b**) horizontal main pattern for 40%; (**c**) horizontal main pattern for 43%; (**d**) horizontal main pattern for 46%; (**e**) horizontal main pattern for 49%; (**f**) horizontal main pattern for 53%.

Fourthly, the gyroid fill has the most complicated printing program. The tracing patterns include main and transitional patterns. As shown in Figure 4a, taking a 36% gyroid fill as an example, a complete cycle of tracing patterns starts from one layer with the transitional pattern. Between 13 layers with horizontal main patterns and 13 layers with vertical main patterns, there is the second layer with the transitional pattern. The third and final layer with the transitional pattern is the end of this cycle and the beginning of the next cycle. For the 13 layers with horizontal main patterns, the patterns of the layers differ only slightly from each other; similarly for the layers with vertical main patterns. The details of each layer tracing patterns in a complete cycle of a 36% gyroid fill are illustrated in Appendix A. The horizontal main patterns of other infill densities are shown in Figure 4b–f.

For the above 19 combinations of infill patterns and densities, a total of 95 standard specimens are prepared. The printed standard specimens are tested by an Instron 3365 Universal Testing System with a 5 kN load cell and a pair of 150 mm-diameter compression platens. The speed of testing is set as 1.3 mm/min, according to D695.

Based on the above test method, for the different infill patterns, if the specimens with the same mass have different compressive strengths, it is not difficult to assume that the different infill patterns have different strength efficiency in providing the compressive property. That is, if one infill pattern has a higher strength efficiency than others, it means the macrostructure inside the specimens based on this infill pattern can provide a higher compressive property with less material. Therefore, including the compressive strength performance of the specimens, the performances of strength efficiency are also compared in the results.

### 2.2. Preparation and Test of Samples of Practical Application

The compressive strength results obtained from the above standard specimen test will be the basis for selecting the proper infill pattern and density for the 3D-printed frame part in the design of a powered ankle-foot prosthesis. The 3D-printed part needs to support the body weight of amputees through a metal adaptor, which is a standard product. Therefore, the structure printed with selected infill pattern and density should provide enough compressive strength and be as light as possible.

**Figure 5:**
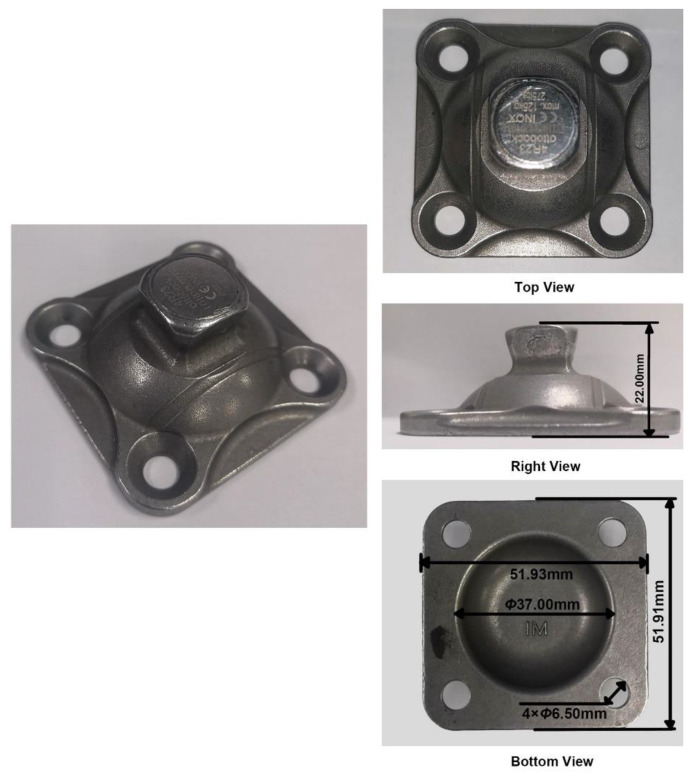
Different views of the 4R23 adaptor.

The selection criterion is decided according to the performance of a standard metal adaptor. The adaptor used in this study is 4R23 (Ottobock, Duderstadt, Germany), whose maximum load allowed is 125 kg. Different views of the adaptor are shown in Figure 5. Firstly, the adaptor will be compressed under the maximum load of 125 kg to determine the corresponding strain. Secondly, according to the measurement from the bottom view of the adaptor, the contact area between the adaptor and the 3D-printed supporting part is 1437.44 mm^2^. If the maximum load is kept at 125 kg, the compressive strength of the 3D printed part should be at least 0.85 MPa at a similar strain, which is the selection criterion for the infill pattern and density.

**Figure 6:**
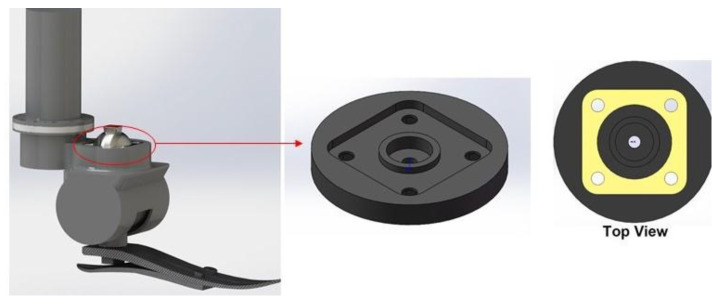
3D model of 3D-printed supporting part.

Compared with the complete conceptual design of the frame part reported in our previous study [35], the structure is simplified only to contain the main functional supporting part related to the assembly of the adaptor, as shown in Figure 6. In the top view, the contact area between the adaptor and the 3D-printed part is highlighted in yellow. 5 samples of the simplified supporting part are printed independently with the selected infill pattern and density. Each sample will be assembled with the same adaptor and tested under a maximum load of 125 kg. The same equipment is used, and the speed of testing is also set as 1.3 mm/min.

## 3. Results

### 3.1. Results of Standard Specimens

**Figure 7:**
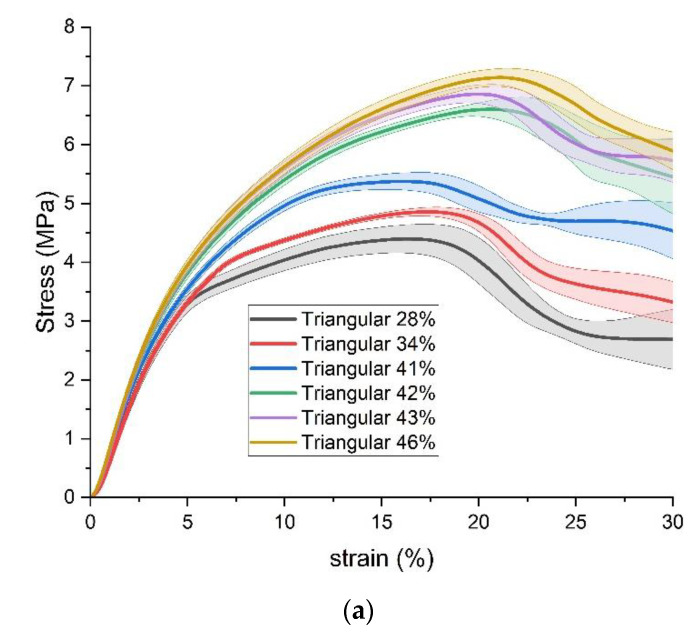

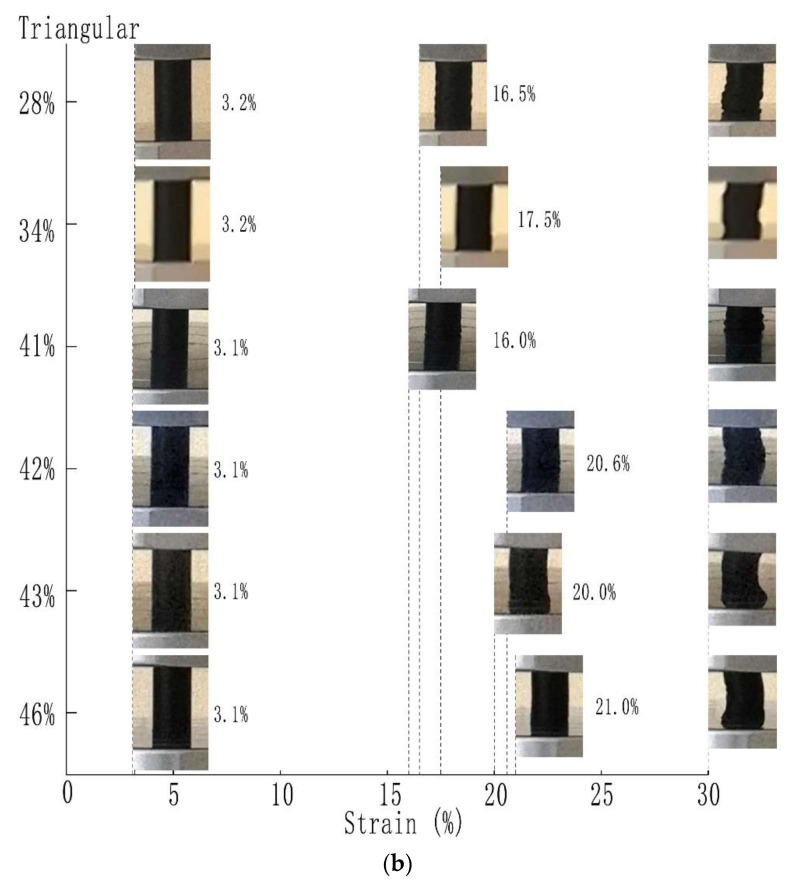
Results of Standard Specimens with triangular fill. (**a**) stress versus strain; (**b**) appearance.

**Figure 8:**
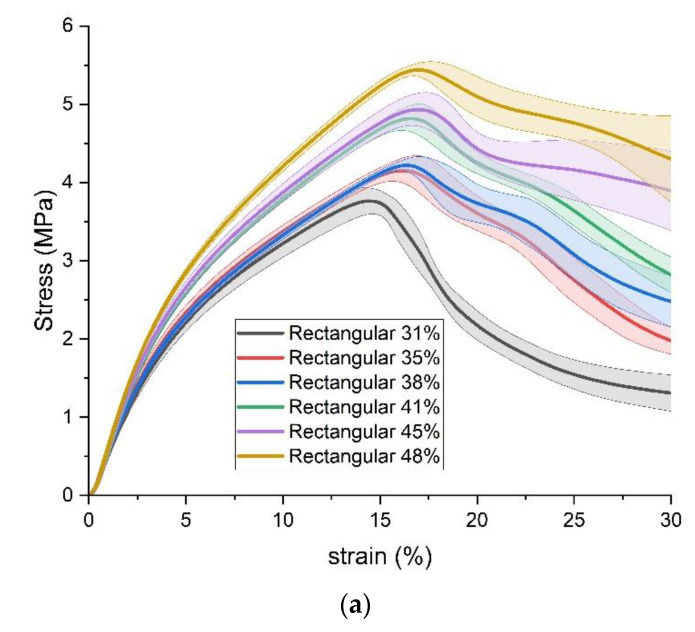

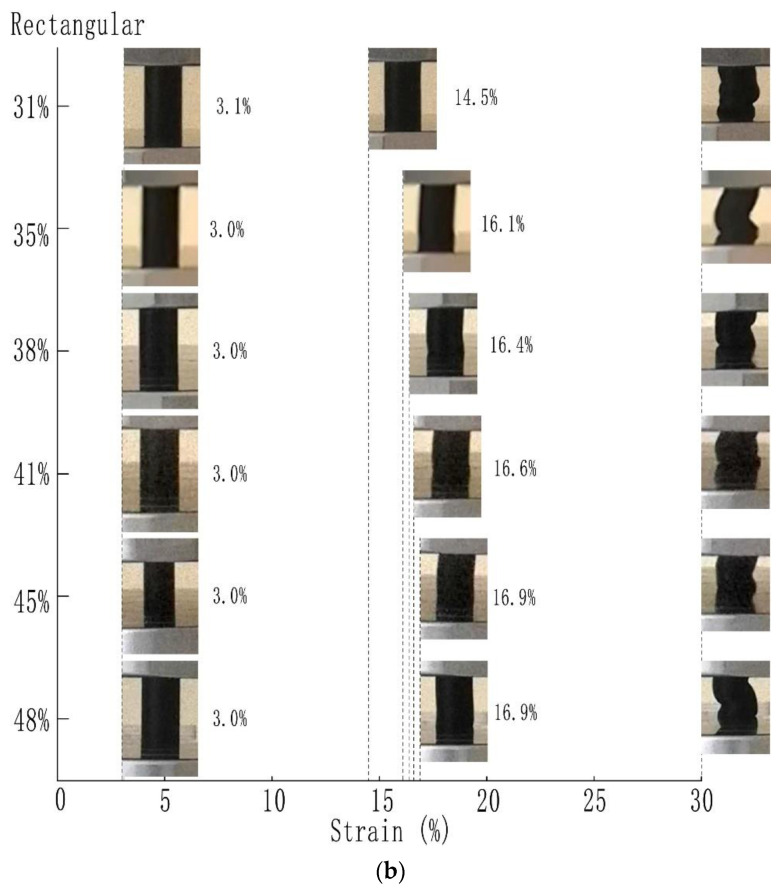
Results of Standard Specimens with rectangular fill. (**a**) stress versus strain; (**b**) appearance.

**Figure 9:**
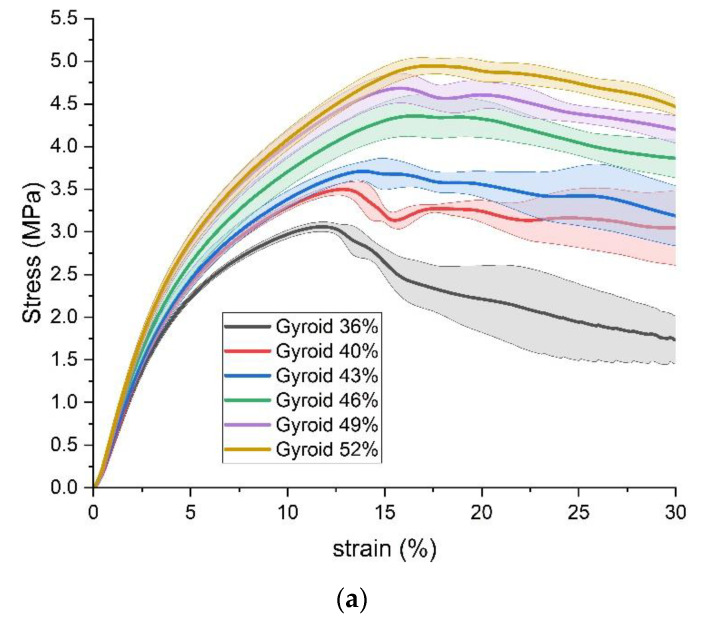

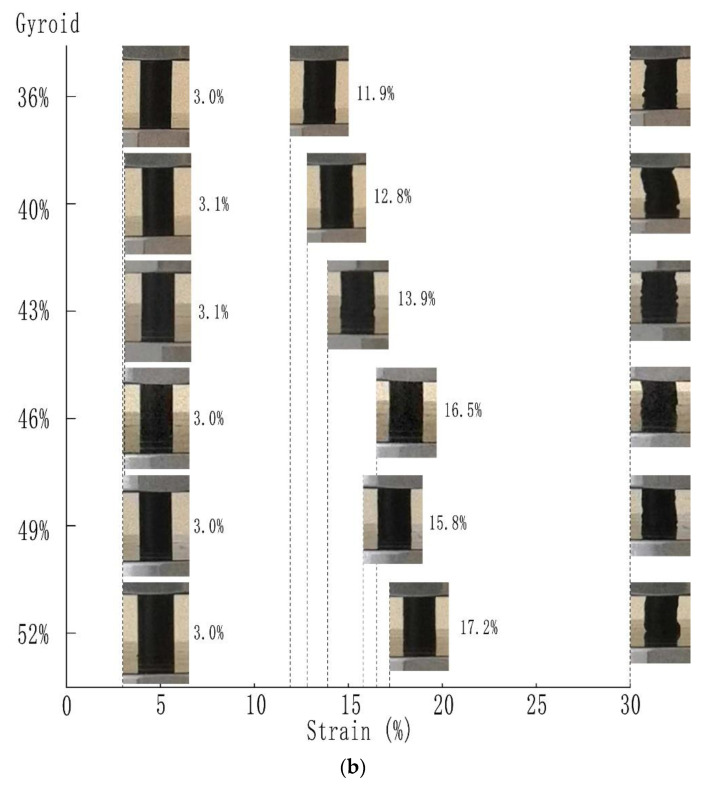
Results of Standard Specimens with gyroid fill. (**a**) stress versus strain; (**b**) appearance.

Firstly, the results of stress, strain, and appearance of standard specimens under the transverse compression load are compared by grouping them with the same infill pattern but different infill densities. The results with triangular, rectangular, and gyroid fills are shown in Figure 7, Figure 8 and Figure 9, respectively. In the plots of stress versus strain, the thick lines illustrate the average results from each group of five specimens with the same 3D printing parameters, and the corresponding shadows are the value ranges of the average plus/minus one standard deviation. Based on the aforementioned average results, the compressive yield strength is estimated by the method of 0.2% offset. In addition, the typical appearances of specimens are compared at three strain points, which are the strain points when the average offset compressive yield strength happened, the strain points when the average ultimate compressive strength happened, and the strain points of 30%.

Overall, the stress-strain curves of the three infill patterns show similar trends within and among the groups. All curves of three infill patterns include three stages. The first stage is the elastic deformation stage, where the stress increases rapidly with increasing strain. Then, in the plastic deformation stage, the slope of stress to strain reduces. Finally, after the appearance of the ultimate compressive strength, with increasing strain, the stress decreases or fluctuates.

For all specimens, the elastic deformation stage stops at a strain point of about 3%. The plastic deformation stages of the three groups end with the appearance of the ultimate compressive strength. The ultimate compressive strengths happen in ranges of strain from 16% to 21% in the triangular-fill group, 14.5% to 16.9% in the rectangular-fill group, and 11.9% to 17.2% in the gyroid group, respectively. After the appearance of the ultimate compressive strength, with the triangular and rectangular fills, with strain increasing to 30%, the stress decreases by more than 1 MPa. However, this decrease in the gyroid fill group is slighter, at about 0.5 MPa.

In each group of infill patterns, as expected, the compressive strength enhances with an increase in the infill density, that is, an increase in the mass of specimens. The values of strain where the ultimate compressive strength appears also possess a gradually increasing trend.

From the perspective of appearance, at the strain points when the average offset compressive yield strength happens, all standard specimens with different infill patterns and densities can maintain the shape of a standard cylinder. When the ultimate compressive strength appears, three kinds of obvious deformation happen on the appearance of specimens. For example, the shape of a wavy line can be found in the front view of the cylindrical surface specimens with 28%, 41%, and 42% triangular fill. An incline of some degrees from top to bottom happens in specimens with 35%, 38%, 41%, and 45% rectangular fill. The example of the specimen with 43% gyroid fill shows that a section near the bottom has an offset away from the radial axis, which looks like a step. When the strain reaches 30%, the degree of the deformations becomes much more severe, and all the abovementioned kinds of deformations also coexist in one specimen.

**Figure 10:**
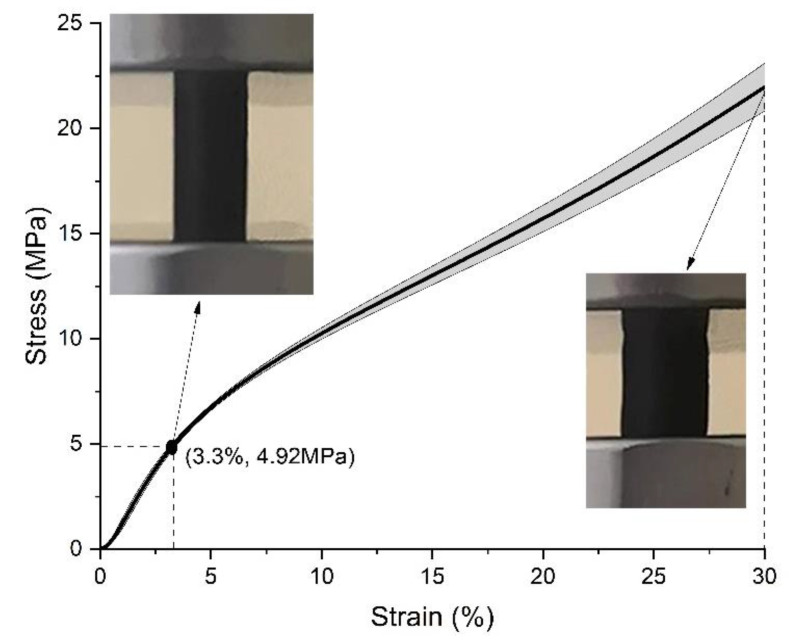
Compression test results of specimens printed with a solid fill.

Secondly, the result of the specimens printed with a solid fill is shown in Figure 10. Similarly, the plot includes the average plus/minus one standard deviation and the typical appearance of specimens at the strain point when the average offset compressive yield strength happened and that of 30%. This result also works as the reference for further comparing the results of standard specimens with similar mass but different infill patterns.

There is a different trend of the stress-strain curve in the solid-fill specimens compared with the above three patterns. In the beginning, the solid-fill specimen is also in the elastic deformation stage, with the stress increasing rapidly with increasing strain, followed by the plastic deformation stage, where the speed of stress increase reduces. However, the ultimate compressive strength does not appear with further increasing strain. That is, the plastic deformation stage is sustained. In addition, from the appearance at 30% strain, the specimen shows some deformation, but its degree is much lower than those of the patterned-infill groups.

**Figure 11:**
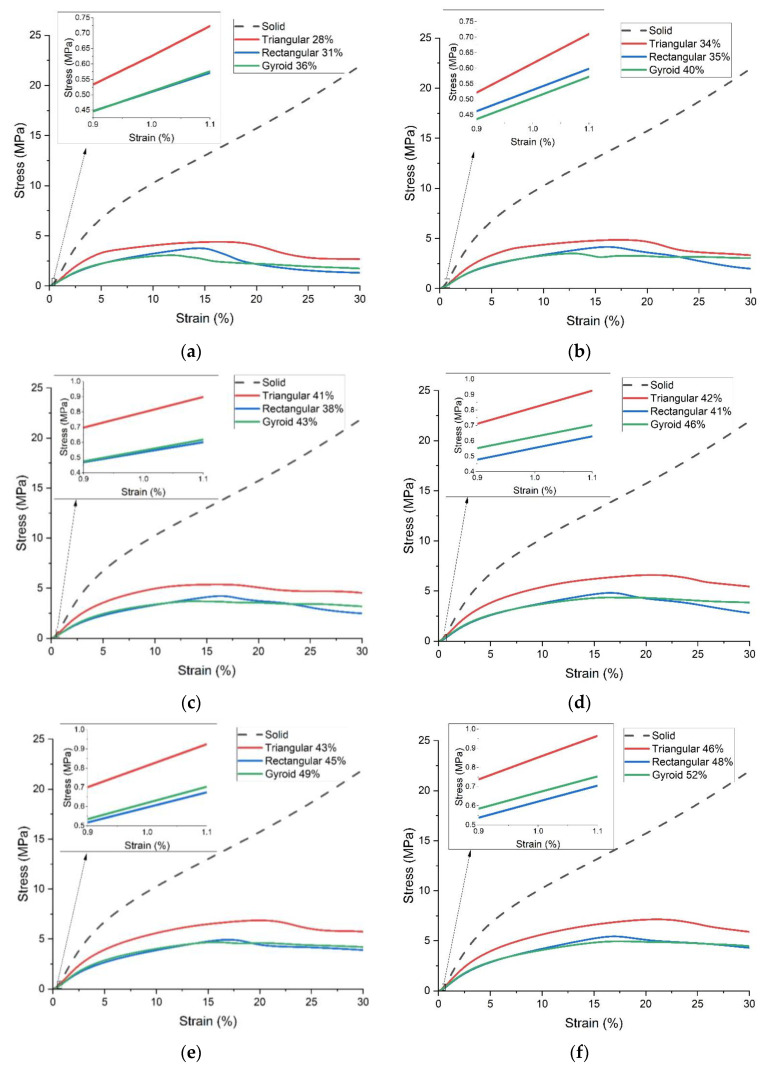
Comparison of compressive strength of different infill-pattern standard specimens grouped by mass. (**a**) 1.6 g; (**b**) 1.7 g; (**c**) 1.8 g; (**d**) 1.9 g; (**e**) 2.0 g; (**f**) 2.1 g.

Thirdly, according to the mass values listed in Table 1, all the above results are compared again, as shown in Figure 11.

It can be seen that the triangular fill performs best among the three patterns in each mass group. There is no considerable difference for the other two patterns in each group, except the ultimate compressive strengths of the rectangular fill are slightly more than the counterparts of the gyroid fill. In addition, for specimens with different infill patterns and densities, weight percentages and compressive property percentages at some strain points within the elastic deformation stage are calculated and compared with respect to the solid specimens, whose mass and compressive strength are regarded as 100%. The results are listed in Table 4.

Focusing on the compressive strength percentage, the values of all three infill patterns show a decreasing trend with the increase in strain within the elastic deformation stage. However, the extents of the decrease are different. In comparing the compressive strength percentage and the weight percentage, in the table, the results are highlighted in which the compressive strength percentage is higher than the corresponding weight percentage. Specimens with the triangular fill can always provide a higher compressive strength percentage than their weight percentage compared with those with the solid fill. However, some specimens with rectangular and gyroid fills can only achieve similar performance at 0.5% strain.

### 3.2. Results of Samples of Practical Application

**Figure 12:**
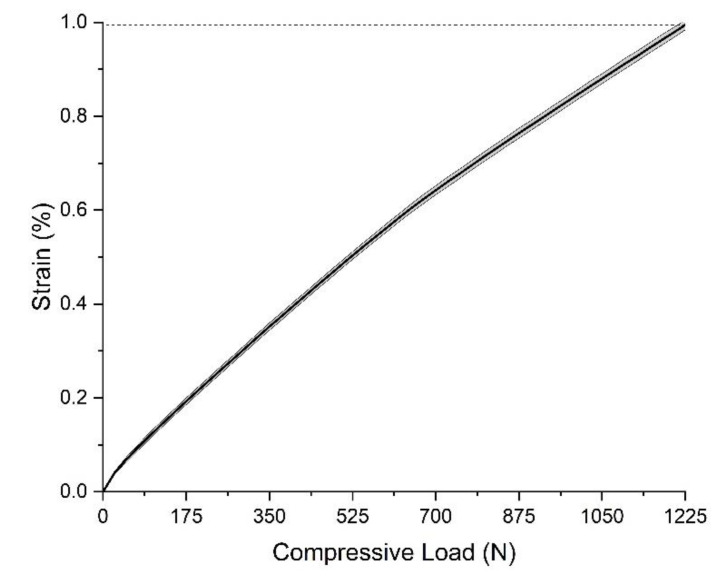
Strain of 4R23 adaptor under the transverse compressive load.

The compression test result of the 4R23 adaptor under the compressive load, of which the maximum is 1225 N, is shown in Figure 12. It shows that the strain is about 1.0% when the compressive load is 1225 N, which is set as the selection criteria of the 3D-printed part.

**Figure 13:**
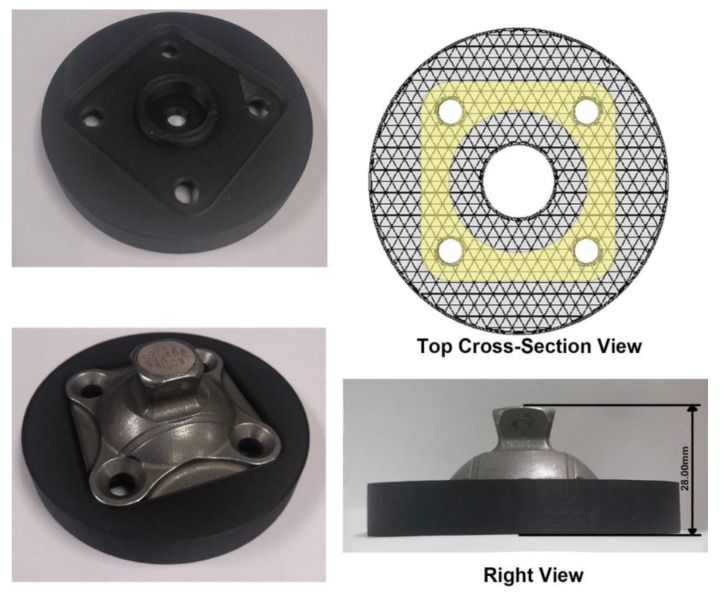
Details of the supporting part and the assembly with the adaptor.

According to the above selection criteria and the partially enlarged details in Figure 11, the specimens printed with the triangular fill with 41% and above infill density can stand 0.85 MPa when the strain is about 1.0%. In this case, we select the lightest infill parameters for the application of the powered ankle-foot prosthesis, that is, 41% triangular fill. Therefore, five samples of the supporting part were printed with the selected parameters. Figure 13 shows the details of the printed samples and those of the assembly of the adaptor and the supporting part. In the top cross-section view, the structure covered by the contact area can be seen. It is worth noting that the assembly does not include any screws and nuts.

**Figure 14:**
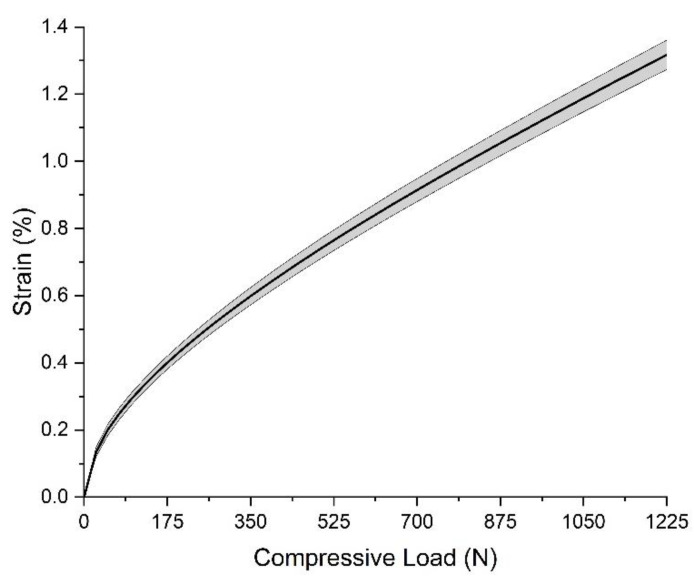
Strain of the assembly of the supporting part and the adaptor under the transverse compressive load.

The compressive test result of the assembly is illustrated in Figure 14. When the compressive load is 1225 N, the strain of the five assemblies is 1.32 ± 0.04%.

## 4. Discussion

The first purpose of this study is to study the effect of infill pattern and density on the compressive strength of 3D-printed Onyx^TM^ material. Because of the vague definition of infill density from the official slicing software, this study employs the mass of standard specimens with different infill patterns and densities as the basis for grouping specimens. For the investigated infill patterns, the rectangular and gyroid fills provide a good linear relationship between the mass of the specimen and infill density, where the mass of the standard specimen increases by about 0.1 g when the infill density increases by about 3% or 4%. However, the linear relationship between the mass and the infill density in the triangular fill is not as good as in the others. Especially when the mass of the specimen is between 1.8 g to 2.0 g, it is susceptible to a change in infill density. Meanwhile, comparing the tracing pattern near the wall in the triangular fill with those in the other two fills, from Figure 2, Figure 3 and Figure 4, it is easy to find that the former is not as regular as the other two. Because of the small cross-section of the standard specimen, the irregular parts of the tracing pattern are crucial, resulting in the high sensitivity of the mass of specimens to the triangular infill density.

In addition, during specimen preparation, there is a common issue in current kinds of slicing software. That is, when the 3D model is imported into the software, the position of the model, such as in the center of the platform or not, will lead to the difference in the inside infill structure. In this case, even though the part that will be printed keeps the same outside size and shape, the tracing pattern is different. The main reason for the difference comes from the slicing process and G-code, which are used to drive the extruder of the 3D printer. G-code includes the motion commands, which are relative to the origin of the coordinate system. The slicing process usually possesses the optimization process to generate the most effective motion commands [36,37]. Accordingly, due to the different relative positions of the target part to the origin of the coordinate system, the optimal result of the infill structure is also different with the same infill parameters. To obtain completely identical specimens, this study printed the identical specimens five times one after the other in the center of the platform instead of printing five specimens at the same time spread across the platform. However, if the technology of 3D printing is used for mass manufacturing, the mechanical properties of the products printed in as many positions as possible should be measured to avoid unreasonably low quality.

There are several facts worthy of note on the stress-strain curve of standard specimens under the transverse compressive load. Firstly, according to the principle of 0.2% offset, the strain points when the offset compressive yield strength happens are around 3% for all infill patterns, including the solid fill. Therefore, there is an assumption that the phenomenon of yield is caused by some structural features possessed by all the infill patterns instead of the difference among infill patterns. The interlayer structure may be the above feature. Because of the inter-bead voids between layers generated in the 3D printing process [38,39], the individual beads can be compressed to occupy the space of the inter-bead voids under the transverse compressive load. This stage should perform as elastic deformation. Once the size of the inter-bead voids is not enough for further deformation, the compressive load will start to break other structure features. That is, plastic deformation happens. The verification of this assumption needs further study.

Secondly, the plastic deformation should be caused by the relative motion of each layer and the intra-layer failure, although the failure of the macrostructure has different patterns of manifestation in the appearance of different specimens. The shape of a wavy line is caused by several small groups of layers that have an interleaved motion to each other. The incline is because all the layers have a motion toward the same direction compared with the adjacent layers. If a large group of layers has a radial motion, but the relative motion in the group is minimal, it will generate an appearance that looks like a step. Because there is much space in each layer consisting of non-solid fills, the material of the upper layer will fall to the lower layer when the interlayer relative motion reaches a certain extent. The falling of material results in the original infill structure being broken. Consequently, the compressive strength starts to reduce after the ultimate compressive strength is reached.

In the group with non-solid fills, the strain points when the ultimate compressive strength happens show a gradually increasing trend with the increase in infill density, which can support the above analysis. Because of the increase in infill density, the space in each layer becomes smaller, which makes the falling of upper-layer material more difficult. Meanwhile, due to the structure of the solid fill, there is almost no space for the falling of upper-layer material. Therefore, the ultimate compressive strength does not show on the stress-strain curve of specimens with the solid fill, even at 30% strain. In addition, for the three non-solid fills, after the ultimate compressive strength is reached, the compressive strength reduces with further increasing strain. However, the degree of reduction with the gyroid fill is smaller than with the other two patterns, which may be caused by the broken inside structures generating new supports for each other during the failure.

On the other aspect, the comparison of weight percentage with compressive strength percentage is important. As mentioned above, if defining a concept of strength efficiency of the 3D-printed structure and regarding the solid fill as the standard, that is, in solid fill, a unit mass of material can provide a unit compressive strength, the efficiency of the triangular fill is higher than that of the solid fill. Even though the solid fill is a special case of rectangular fill, not only cannot the efficiency of the rectangular fill reach that of the solid fill, but it is also lower than that of gyroid fill. It is also worth noting that the strength efficiency of different infill patterns does not vary linearly with infill density. That is, there is a moderating effect of infill patterns on the influence of infill density on the mechanical property.

Finally, regarding the application related to the powered ankle-foot prosthesis, the average performance of the assembly of the standard adaptor and the 3D-printed supporting part is slightly worse than the standard compressive specimens. The reason is that there is no structure of wall included below the contact area, which reduces the compressive strength to some extent. However, it is acceptable that the difference of 0.32% strain under 1225 N exists for the 3D-printed frames of powered ankle-foot prostheses.

## 5. Conclusions

This study investigates the effect of infill parameters on the compressive strength of 3D-printed carbon fiber-reinforced nylon material. Two infill parameters are studied. One is the infill pattern, including triangular, rectangular, and gyroid fills. The other is the infill density, which is transferred into the mass of standard compression specimens based on each infill pattern. The results show that the triangular fill is the best for compressive strength, and the more mass the specimen has, the higher the compressive strength is. A 41% triangular fill is selected to manufacture a supporting part for the powered ankle-foot prosthesis. Under the required compressive load, the strain of the assembly of the standard adaptor and the 3D-printed part can meet the requirement of the design of powered ankle-foot prostheses. The results of this study can benefit the adoption of 3D printing technology in more applications that have a specific requirement of compressive strength and low weight of 3D-printed parts.

## Figures and Tables

**Table 1:** Summary of previous literature related to infill density.

Reference	Parameters	Materials	Mechanical Properties
[9]	infill density; layer thickness; raster angle	PLA	flexure strength
[10]	infill density; build orientation	ABS	tensile strength
[11]	infill density; build orientation	PEEK	tensile strength
[12]	infill density; layer thickness; raster angle	ABS	tensile strength
[13]	infill density	PLA	tensile strength, impact strength
[14]	infill density; layer thickness	PLA-graphene	tensile strength; impact strength; flexural strength
[15]	infill density; layer thickness; extruder temperature	PLA	tensile strength
[16]	infill density; layer thickness; build orientation; extruder temperature	PLA	tensile strength
[17]	infill density; layer thickness; print speed	Nylon	tensile strength; impact strength; flexural strength

**Table 2:** Summary of previous literature related to infill pattern and density.

Reference	Parameters	Materials	Mechanical Properties
[20]	infill density; infill pattern; perimeters; shell thickness; extrusion multiplier	ABS	tensile strength
[21]	infill density; infill pattern; raster angle	ABS	tensile strength
[22]	infill density; infill pattern; layer thickness; nozzle diameter	Carbon Fiber-reinforced Nylon	tensile strength; impact strength; flexural strength; hardness
[23]	infill density; infill pattern	PLA	tensile strength
[24]	infill density; infill pattern; layer thickness	ABS	tensile strength
[25]	infill density; infill pattern; build orientation	PLA	tensile strength
[26]	infill density; infill pattern	PLA	tensile strength; flexural strength;
[27]	infill density; infill pattern; build orientation	PLA	tensile strength
[28]	infill density; infill pattern	PLA	tensile strength

**Table 3:** Mass of compressive specimens with different infill patterns and densities.

Mass (g)	Weight Percentage (%)	Infill Patterns		
		Triangular	Rectangular	Gyroid
1.6	45.71%	28%	31%	36%
1.7	48.57%	34%	35%	40%
1.8	51.43%	41%	38%	43%
1.9	54.29%	42%	41%	46%
2.0	57.14%	43%	45%	49%
2.1	60.00%	46%	48%	52%
3.5	100.00%	Solid		

**Table 4:** Comparison of weight percentage versus compressive property percentage.

Infill Patterns and Densities	Weight Percentage	0.5% Strain	1.0% Strain	1.5% Strain	2.0% Strain	2.5% Strain
Solid	100% (3.5 g)	100% (0.39 MPa)	100% (1.22 MPa)	100% (2.14 MPa)	100% (3.02 MPa)	100% (3.82 MPa)
Triangular 28%	45.71%	52.27%	51.48%	51.46%	51.06%	50.74%
Rectangular 31%		53.27%	41.91%	37.80%	35.75%	34.61%
Gyroid 36%		50.39%	42.05%	38.70%	37.06%	36.04%
Triangular 34%	48.57%	49.28%	50.69%	50.94%	50.91%	50.76%
Rectangular 35%		50.55%	43.63%	40.20%	38.36%	37.22%
Gyroid 40%		46.74%	41.50%	39.29%	38.25%	37.62%
Triangular 41%	51.43%	78.73%	65.64%	60.43%	57.97%	56.46%
Rectangular 38%		54.76%	44.07%	39.80%	37.63%	36.36%
Gyroid 43%		52.41%	44.98%	41.65%	39.87%	38.80%
Triangular 42%	54.29%	79.34%	67.36%	63.13%	61.14%	59.92%
Rectangular 41%		48.79%	45.62%	43.08%	41.55%	40.56%
Gyroid 46%		66.85%	51.62%	46.26%	43.70%	42.21%
Triangular 43%	57.14%	73.87%	66.88%	63.78%	62.22%	61.23%
Rectangular 45%		55.28%	48.99%	45.45%	43.42%	42.18%
Gyroid 49%		54.11%	50.93%	48.21%	46.69%	45.61%
Triangular 46%	60.00%	79.74%	70.13%	65.63%	63.37%	62.02%
Rectangular 48%		54.87%	51.20%	47.91%	45.98%	44.78%
Gyroid 52%		66.47%	55.12%	50.26%	47.76%	46.28%

**Figure A1:**
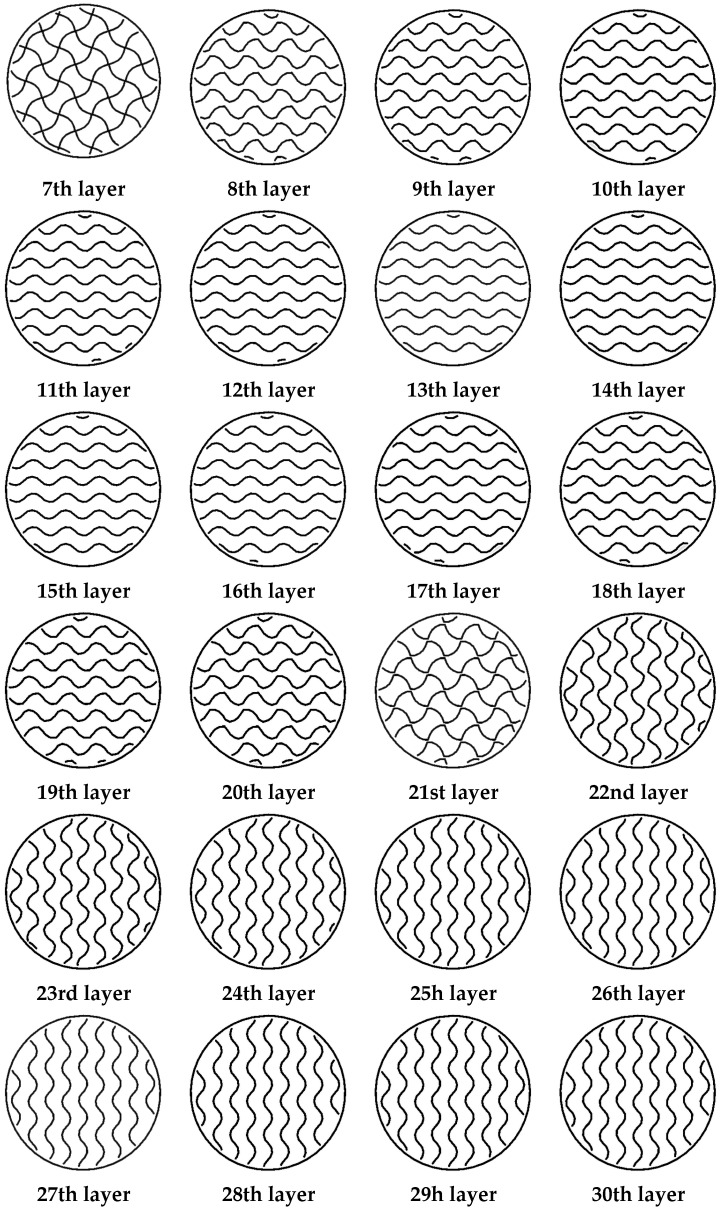

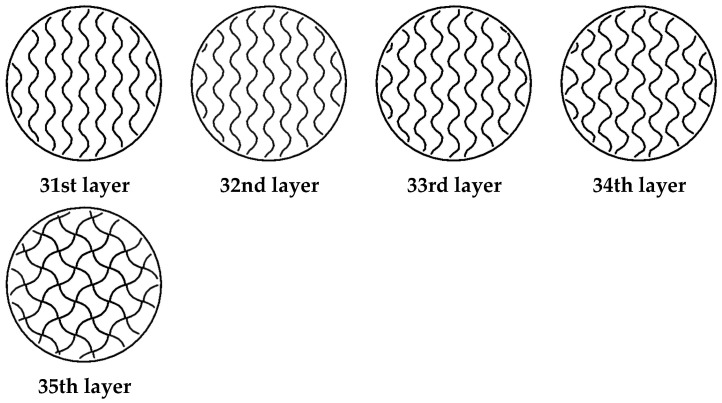
Tracing pattern in each layer of a 36% gyroid fill in a complete cycle from the 7th layer to the 35th layer.

## Data Availability

Not applicable.
